# An algorithmic approach to scalp reconstructive surgery: maximization of cosmetic and functional outcomes

**DOI:** 10.1007/s00403-024-02896-3

**Published:** 2024-04-29

**Authors:** Mustafa Mahmood, Daniel Eisen

**Affiliations:** 1 Albany Medical College, Albany, NY USA; 2 Department of Dermatology, University of California Davis Medical Center, Sacramento, CA USA

**Keywords:** Scalp reconstruction, Skin flaps, Algorithmic approach

## Abstract

Background: Scalp reconstruction requires knowledge of scalp anatomy and reconstructive options. Advances in the field have led to numerous procedures being at the disposal of the reconstructive surgeon, expanding treatment options for patients. Objective: To provide an algorithmic approach and general guidelines to consider when deciding on which scalp surgery will optimize cosmetic and functional outcomes. Methods & materials: Previous literature was searched for the last 20 years to provide an updated guide. Results: Taking into consideration the location, size and local scalp anatomy of a presenting defect will lead to optimal surgical outcomes. Other confounding factors such as bone exposure and extremely large defects will affect decision making. An algorithmic approach has been provided in this review. Conclusion: While many reconstructive surgical options are available, the best ones will depend on individual presentation of scalp defects. Location and size are first line considerations while local scalp anatomy will allow for tailoring of reconstructive options. This will help to maximize cosmetic and aesthetic considerations.

## Introduction

Scalp reconstruction is often difficult: the variable mobility of the scalp, often inelastic nature of the tissue, as well as the scalp’s convex shape all contribute to this issue. Achieving optimal cosmetic results adds to the burden of reconstructive surgeons as care must be given to preserving the hairline, minimizing scars, incision lines, alopecia and any other abnormalities that may arise. Nonetheless, current advances in the field have resulted in a number of options to allow reconstructive surgeons to best optimize functional and aesthetic results.

**Fig. 1:**
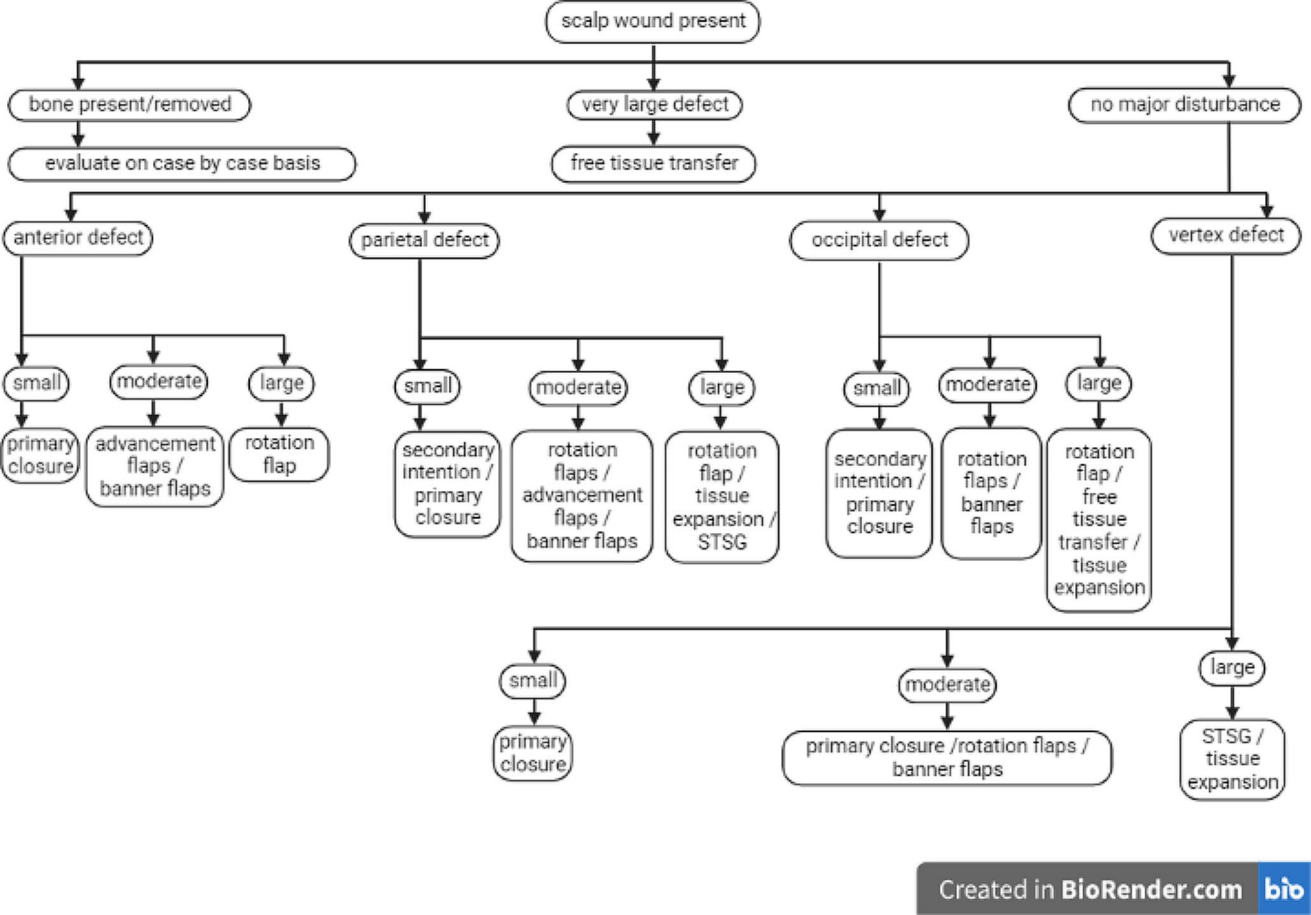
An algorithmic approach to evaluate scalp defects based on size and location that presents the best overall cosmetic and functional outcome

This review will highlight the major general guidelines and provide an algorithmic approach to maximize cosmetic and functional restoration of the scalp (Fig. 1).

### Anatomy and physiology

#### Layers

The layers of the scalp, from superficial to deep, are the skin, sub-cutaneous tissue, aponeurotic layer (or galea), loose areolar tissue and the pericranium.

The skin is the most superficial layer. Underneath the skin lies the subcutaneous layer comprised of fat, blood vessels, nerves and lymphatic vessels that course throughout the scalp [2, 13, 22].

Deep to the subcutaneous tissue lies the galea which confers the strength of the scalp due to its connection with the facial musculature. The galea is continuous with the frontalis muscle anteriorly, the occipitalis muscle posteriorly and temporoparietal fascia laterally. It fuses with the pericranium at the Linea temporalis and conjoint tendon. The galea is also relatively inelastic due to the high confluence of tissue which makes some flaps more difficult to perform.

Underneath the galea lies loose areolar tissue which is responsible for most of the scalp’s mobility. This mobility is not uniform throughout the scalp and is greatest in the parietal regions. It significantly decreases in the superior temporal septum in the lateral frontal region due to the adherence of the areolar tissue with the pericranium. Additionally, this region represents the confluence of the frontal periosteum, the deep temporal fascia and the temporoparietal fascia [2, 13, 22].

The deepest layer of the scalp is the pericranium which constitutes the periosteum of the underlying cranial bone. The pericranium should be left intact, when possible, to facilitate wound healing, graft survival, and decrease chances of air embolism [13, 22].

#### Vascularity

An understanding of the scalp’s vasculature is critical. Anterior regions are perfused with the supratrochlear and supraorbital arteries, the terminal branches of the internal carotid. Lateral areas are supplied by the superficial temporal artery and posterolateral areas are perfused by the posterior auricular artery, both branches of the external carotid. Posterior sections are supplied with different vessels based on their relation to the nuchal line. The area above the nuchal line is supplied by the occipital artery and below the line are the branches of the trapezius and splenium capitis arteries [2, 22].

#### Innervation

The scalp is innervated by each of the three divisions of the trigeminal nerve as well as the cervical spinal nerve and the cervical plexus. Superficially in the ophthalmic division of the trigeminal nerve, the supratrochlear nerve, and the supraorbital nerve innervates the forehead, the frontoparietal scalp, and the anterior hairline. From the maxillary division, the zygomaticotemporal nerve innervates the region lateral to the brow up to the superficial temporal crest. In the mandibular division, the auriculotemporal nerve innervates the lateral scalp area. The occipital region is innervated by both the greater occipital nerve from the cervical spinal nerve and the occipital nerve from the cervical plexus [2, 13, 22].

### Reconstructive guidelines

Due to the wide variety of patient history and clinical presentation of scalp defects, utilization of the reconstructive ladder provides surgeons with a method to determine the best procedure for each patient. The ladder proceeds in the following manner: secondary intention, primary closure, advancement flaps, rotation flaps, skin grafts, tissue expansion, and free tissue transfer [4]. The ladder begins with simpler procedures, increasing in complexity further up the ladder [7, 9]. The best procedure is one that is simple, durable and provides the best cosmetic results.

Since scalp tissue is unique due to its underlying structures and hair-bearing patterns the best replacement for scalp tissue is scalp tissue. Therefore, local redundant tissue will best allow for functional restoration and maximize cosmetic outcomes by mimicking local skin coloration, texture, shape and hair patterns. This will reduce the incidences of alopecia, color mismatch and height discrepancies which is seen when local tissue is not used [7, 13].

For this review, small defects are defined as < 3 cm in diameter, moderate as 3–5 cm and large as > 5 cm.

## Guidelines based on location and defect size

### Anterior defects

The anterior of the scalp is a cosmetically sensitive area. Preservation of the hairline and camouflaging incision scars are important considerations. When planning the closure of the wound, tension should not be placed on the skin itself which could result in alopecia and dehiscence. Instead, tension should be focused on the galea as it is deep to the hair follicles and can withstand the added force of closure [4, 13]. Skin grafting and free tissue transfer should be avoided, unless there are no other options, due to poor cosmetic results.

#### Small

Small defects are amenable to be treated with primary closure [4]. Secondary intention could be used in this circumstance though this is best suited for those with androgenetic alopecia, where the scars are less conspicuous, than for those who still have hair.

#### Moderate

For moderate wounds, where primary closure is not feasible, advancement flaps and rotation flaps may be considered. The H flap is a great option and requires less surgical work than rotation flaps [4]. It has also been documented that an A-T flap could provide great results for anterior defects due to the flap needing less mobility. Additionally, the basal scar of this flap can be camouflaged with the rest of the forehead lines [12].

#### Large

For these defects, large rotation flaps may be used, provided that the hairline is not involved. This will provide the best cosmetic and functional result in this region of the scalp [13].

### Parietal defects

The parietal region of the scalp is not cosmetically sensitive. Scars can be well hidden with hairstyling and hats. Tissue in this region is very mobile which allows for a wider variety of reconstructive options to be considered [13].

#### Small

Small wounds can be covered using secondary intention or primary closure, due to the higher scalp mobility here [11]. Reportedly, rhombic flaps are a good option as they help to maintain the hair pattern between the lateral and parietal scalp [3, 13].

If using secondary intention, healing time ranges from 6 to 8 weeks but might be longer if the periosteum is absent [2]. This is best used in patients who want minimal surgery, have multiple comorbidities resulting in disqualification from other surgeries, wounds that are too large to be closed by other methods, and who have baseline alopecia. This technique allows for little surgery time and is usually less painful than other reconstructive options. Very few contraindications are present [2].

#### Moderate

For moderate defects, rotation flaps are a great option. Options include O to Z flaps, rhombic flaps and ortichea flaps. It is best to use rotation flaps that place the standing cones posteriorly opposed to laterally for optimal cosmetic outcomes. Galea scoring could also be helpful [13, 21]. Attention to flap design must be observed to preserve the hairline as best as possible [5, 13, 21].

Advancement flaps are another option. H-plasty has been described to yield good results for small to moderate sized defects in the posterior region of the scalp. They also have smaller incision scars and are easier to design than rotation flaps. The larger incisions of rotation flaps combined with their sweeping nature comes with more reported numbness compared to H-plasty. Nonetheless, both remain great options [10].

Skin grafts could also be considered for this region although alopecia, color mismatch and height discrepancy are all likely to occur [7, 9].

#### Large

For large defects, interpolation flaps like the juri flap or posterior cervical rotation flaps can be considered [6]. Modified advancement flaps could also be of use. V to Y advancement flaps that use the occipital artery for perfusion have been shown to provide excellent cosmetic results. The flap utilizes local redundant tissue and minimizes the presence of dog ears. This flap design is purportedly easier to perform compared to large rotation flaps or double flaps [20].

Tissue expansion could also be considered in conjunction with skin grafting. Utilization of tissue expansion is a staged procedure where the expander is placed in the subgalea area [4]. Multiple follow up visits are required to ensure adequate tissue expansion and assess any potential complications [2, 15]. Tissue expansion requires several months and incurs a significant psychosocial burden due the obvious deformity that is present during expansion. Some complications, like pain on expansion, are minor and can be resolved without changing the reconstructive approach. Other major documented complications, like infection, exposure of the expander and bone erosion, necessitate a change of plans and are known to occur frequently [14, 18].

### Occipital defects

**Fig. 2:**
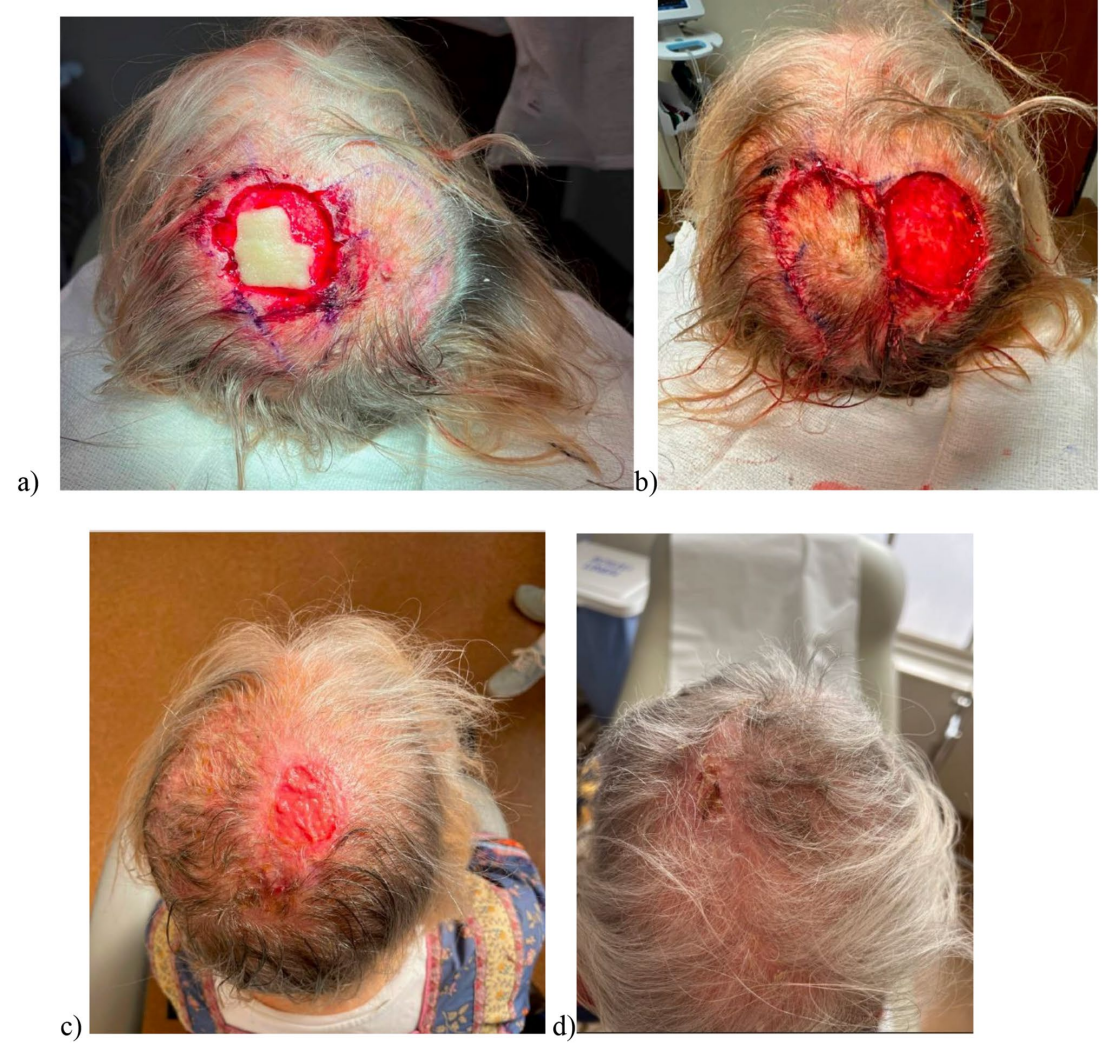
Progression of a banner flap on occipital scalp from **(a)** pre-operative flap design, **(b)** flap during surgery, **(c)** flap immediately after surgery and **(d)** flap healed by secondary intention following 1–2 month follow up

The occipital area is also of low cosmetic pressure; therefore, defects can be covered with hair styling or clothing (Fig. 2).

#### Small

As with most other small defects, primary closure or secondary intention are great options to consider. Dog ears are not a large concern in this area [21].

#### Moderate

Moderately sized wounds are amenable to rotation flaps. There is a moderate amount of mobility in the occipital region that lends ease to different rotation flaps that use local tissue. Care should be given in order to preserve the posterior hairline as much as possible [21].

#### Large

Larger defects would require large scale rotation flaps, like the ortichea flap, free tissue transfer or occipital artery V to Y flap. It is possible to use tissue from the local trapezius muscle for occipital cervical reconstruction; however, it has been reported that this procedure has resulted in cases of wound breakdown and multiple follow up visits that extend operating times [24]. Tissue expansion has shown excellent cosmetic results in this region alongside skin grafting for qualifying patients [21].

### Vertex defects

The vertex of the scalp is the most immobile areas due to the heavy presence of galea with no underlying musculature. Therefore, it is difficult to perform many techniques that are otherwise easy to perform in other areas of the scalp [4].

#### Small

Small defects are best treated with primary closure. If that is not possible, then small rotation flaps, like pinwheel or rhombic flaps, are an option [4].

#### Moderate

For moderate vertex scalp defects, primary closure with increased undermining is possible. Triple and modified quadruple rhombic flaps work well and maintain the natural spiral of the hairline [17, 4].

#### Large

For larger defects, rotation flaps can be an option, however they would require near total scalp undermining which would increase their difficulty in performing. For this reason, tissue expansion is often considered along with free tissue transfer [4].

## Other reconstructive circumstances

### Removed bone

When tumor invades beyond periosteum, many will consider referral to a head and neck surgeon, though some Mohs surgeons continue to use the Mohs chemo paste to address these situations. When most of the soft tissue, periosteum and, occasionally, cranial bone and dura, are removed reconstructive options must be carefully considered. The specific circumstances of the surgical defect will inform what reconstructive procedures can be performed [2, 4, 5, 21].

When the outer table of bone is removed, it can heal by second intention, but is often managed with skin grafting, which reduces the risk for cerebral air embolus, that can occur when non-collapsible diploic veins are exposed to air [8, 23].

Rotation flaps can be performed with absent pericranium which is helpful for patients who have deep tumor excisions. Free tissue transfer is also viable and the donor site may be covered with a skin graft [2, 4, 5, 21].

Titanium implants can be a potential option to replace the removed bone. They are the most rigid compared to other materials and have anti-inflammatory and anti-bacterial properties [16].

In patients with moderate to large sized defects and exposed bones, a combination repair using a banner flap and a split thickness skin graft (STSG) has shown effective. In this procedure, the banner flap allows for closure of the primary defect and the STSG covers the secondary defect [1]. This is better than using a STSG for the primary defect since the absence of any tissue above the calvarium makes it difficult for the skin graft to successfully be implemented. Furthermore, this procedure is superior to free tissue transfer due to the better cosmetic outcomes and shorter procedure times as the procedure can be done in one stage with a low complication rate [1].

### Extreme defects (> 10 cm diameter)

**Fig. 3:**
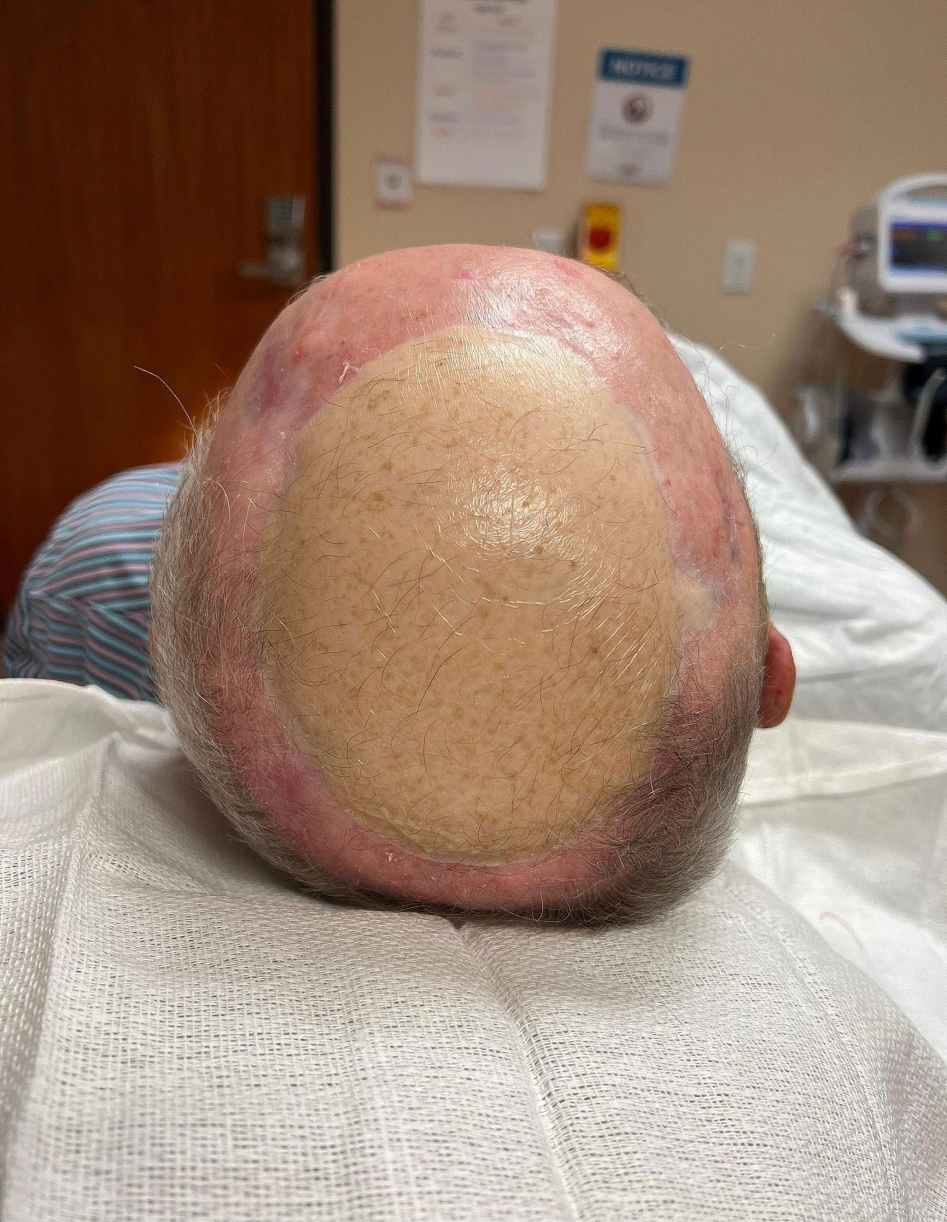
Post operative free flap on parietal scalp highlighting relatively poor cosmetic results in alopecia patients

Free tissue transfer is used for extreme defects, absent periosteum, patients with a history of radiotherapy, osteomyelitis, osteoradionecrosis, prior local flap failure, heavy trauma, high grade malignancy or exposed cranial bone. The best free flaps will have low donor site morbidity, long pedicles, reliable anatomy and versatility in shape. Vascularity is usually not a problem as the pedicles from common donor sites are large enough to properly stabilize the flap and allow for adequate anastomosis. Due to this, it is common to harvest a free flap as a muscle only flap and then place a full thickness skin graft (FTSG) on top in order to maximize cosmetic results, though poor results are common [15] (Fig. 3).

There are five main types of free flaps that will be covered in this review: latissimus dorsi (LD), rectus abdominis (RA), anterolateral thigh (ALT) flaps, radial forearm (RF) and the Omental (OM) flap. The LD and RA flaps are usually muscle only flaps due to the bulk of the subcutaneous tissue that is present in those areas [21].

LD flaps have several advantages: they provide a large amount of tissue, are well suited for microvascular anastomosis, have long pedicles and can be harvested with the local rib to help with cranioplasty if necessary [7, 9, 15]. Additionally, the muscles in the flap naturally contour to the shape of the pericranium. Disadvantages include the multi-surgeon teams, longer OR times due to patient positioning, and patients expressed post-operative reduction in shoulder abilities. Cosmetic outcomes are usually poor and necessitate a STSG [6].

ALT flaps have some advantages over LD flaps. The scarring from removal is easily hidden with clothing, OR times are shorter, they have minimal functional morbidity, and the flap needs less skin grafting. Disadvantages include bulky subcutaneous fat, variable pedicle lengths, inability to cover total scalp defects, and thicker, less pliable tissue. It also migrates caudally over time and the flap could contain hair, for relevant cosmetic considerations [6, 19].

RF flaps are uncommon but work well for defects less than 7 cm. They have reliable and long pedicles, thin pliable skin and allow for easy harvesting and resection. Its main disadvantage is its inability to cover total scalp defects [15, 19].

RA flaps have large pedicles, don’t need additional grafting and the location of the flap allows for easy harvesting and resection. However, there is increased risk of abdominal hernia from harvesting the flap and the tissue is not as pliable [15, 22].

Finally, the OM flap can be used and is well equipped for covering large defects. OM flaps have pedicles which can be extended to 20 cm, allowing for anastomosis with the neck vessels, have short OR times, large amount of soft tissue, high vascularity, minor complications and good aesthetics. However, the flap requires a laparotomy, of which prior abdominal surgery could be a contraindication, and the volume available for transfer differs from patient to patient [6, 15].

## Conclusion

Scalp reconstruction is often difficult. Knowledge of all reconstructive options available for patients will maximize both functional and cosmetic outcomes and reduce morbidity. Due to the complexity of the scalp’s anatomy and physiology, the best treatment options for patients will vary on a case-by-case bases depending on clinical presentation and underlying patient circumstances.

## Data Availability

No datasets were generated or analysed during the current study.
